# The impact of a fast bowling spell on physiological, perceptual and performance responses in non-elite cricketers

**DOI:** 10.17159/2078-516X/2019/v31i1a5624

**Published:** 2019-01-01

**Authors:** L Pote, S Proctor, K McEwan, J P Davy, C J Christie

**Affiliations:** Department of Human Kinetics and Ergonomics, Rhodes University, Grahamstown, South Africa

**Keywords:** accuracy, speed, heart rate, body discomfort, ratings of perceived exertion

## Abstract

**Background:**

The demands placed on fast bowlers may elicit unique responses that contribute towards increased injury risk and comprised performance capabilities. Despite this, very few investigations have attempted to quantify these demands and their impact on performance in cricketers.

**Objective:**

This investigation attempted to quantify the effects of a fast bowling protocol on the musculoskeletal, physiological and perceptual responses of fast bowlers; as well as ball speed and accuracy.

**Methods:**

Eight young adult bowlers (20 ± 2 years) participated in a 10-over bowling protocol that had been separated by intermittent fielding drills into three bowling spells respectively (4-, 3- and 3- overs). Selected responses were collected throughout the protocol.

**Results:**

Functional strength was measured and showed no change. Heart rate responses increased significantly (p<0.05) at the start of the bowling protocol. Local ratings of perceived exertion increased significantly (p<0.05) as a function of exercise duration, while low to moderate intensities of perceived discomfort were noted in the anterior and posterior shoulder areas, upper portion of the lower limb musculature, as well as in the middle and lower back regions. Performance responses experienced no significant change.

**Conclusion:**

There was no significant change in ball release speed and accuracy across the bowling protocol. Lower limb muscle power remained consistent and heart rates reached a steady state after the first over. In comparison, local ratings of perceived effort and body discomfort increased over time, which could mean that those unchanged measures do not accurately reflect fatigue or that perceptions are a more effective indicator of impending fatigue.

Fast bowlers are essential to a cricket team’s success as they assist in restricting the number of runs scored by the opposing team.^[1]^ This is achieved through manipulating factors such as the line, length, lateral deviation and speed of ball delivery.^[1,2]^ The line and length are highly dependent on the batter facing the ball, external weather conditions, as well as the surface of the pitch.^[2]^ Fast bowlers strive primarily to attain high ball speeds (together with accuracy) as this reduces the time available to the batsmen to process the delivery and execute an appropriate motor response.^[1,2]^

The attainment of high ball speeds requires fast bowlers to perform multiple actions simultaneously, in a short period of time (approximately one second), placing immense physical demand on the body.^[1]^ This may negatively affect performance-related parameters, such as ball release speed and accuracy.^[2,3]^ Furthermore, these actions are performed repeatedly and at high intensities, during training and competition.^[4,5]^ As a result, fast bowlers have the highest risk of injury, with prevalence rates of approximately 42%.^[4]^

Despite indirect evidence of the physical demands of fast bowling, very few studies have focussed on identifying and describing these variables.^[3,5]^ A reason for this may be linked to the fact that it is difficult to quantify the loads being placed on the bowler and the resultant responses, because of the irregular ‘stop-start’ nature of fast bowling.^[5]^ Furthermore, care should be taken when interpreting the current available literature/research as it focuses primarily on the acute responses to bowling load, such as heart rate, blood lactate concentrations and hydration status.^[6]^ Only a paucity of evidence exists which describes/compares the relationship between the physiological, biomechanical and perceptual demands of fast bowling and performance in a competitive environment.^[3,8]^ This is despite these factors being found to comprise the cornerstones of the conceptual model (Centre-M model) for studying human movement.^[5]^

Additionally, no study has examined the impact of lower limb fatigue on bowlers despite the fact that in other intermittent sports functional strength was used to investigate this response.^[7]^ Nevertheless, there is a need to obtain data from different calibres of players to corroborate the validity of such findings. Further research that is based on a holistic, interdisciplinary approach is required to deepen the understanding of the physical demands of fast bowling, so that injuries may be prevented and performance improved.^[5]^ Therefore the purpose of this study was to investigate the effects of a fast bowling protocol on the physical, perceptual and performance responses in non-elite fast bowlers.

## Methods

### Study design and participants

This investigation was a repeated measures design that assessed the impact of a fast bowling protocol on selected physiological, perceptual and performance measures over time. Eight male, non-elite first- and second-team school- and university-level fast bowlers, between the ages of 17 and 21 years, were recruited (age and level of experience were controlled). Fast bowlers were defined as those players bowling over 80 km.h^−1^ with a run-up of between 15 and 30 m. The study was approved by the Rhodes University Ethical Standards Committee (Rhodes University, Grahamstown, South Africa) (RU-HSD-15-06-0012) and players were informed of the risks and benefits of the investigation.

### Testing procedure

The testing occurred in two phases, initially including accustoming the cricketers to the testing procedures, as well as the collection of selected baseline, demographic and anthropometric measures. Secondly, cricketers were required to simulate a bowling protocol following a dynamic warm-up focusing specifically on the upper and lower limbs of the body, in addition to the lumbar area of the spine. The bowling protocol was based on a time-motion analysis that determined the time between deliveries, as well as the time taken to switch ends at the completion of the over.^[8]^

Previous research has shown that the average time between deliveries was 39 seconds and the change of ends after each over took approximately one minute and 20 seconds, which were the intervals used in this study.^[8]^ These intervals are similar to those of competitive schoolboy matches determined anecdotally and by the authors’ personal experiences. The change over period was simulated by the player walking around slowly to a specified point on the cricket pitch. After the completion of each changeover, intermittent fielding drills were completed which lasted for three minutes 54 seconds each, while the other bowler did his bowling spell. Fielding drills involved walking ten metres towards the stumps as each ball was delivered by the other bowler.^[3]^ Each player was also expected to run for 20 metres on the second and fourth ball of the over and to field a total of one ball that was randomly completed during the over.^[3]^

Following this, the one minute 20 seconds changeover was once again initiated to allow the players to get ready to begin the next over. Since the maximum number of overs that can be bowled during a 50-over match has been identified as ten which was selected as the number of overs to be bowled for this particular bowling protocol. The specified number of overs to be bowled was further divided into three bowling spells (4-, 3- and 3- overs respectively) since it is very rare for all ten overs to be completed in succession. Once the protocol was complete, each player cooled down with stretches. The stretching session involved static stretches of both lower and upper limbs, as well as any other stretches that the participants felt they needed.

### Measurement and instrumentation

#### Physiological measures

Heart rate was measured throughout the protocol using a PolarTM F11 heart rate monitor (Polar Electro Oy, Kemple, Finland) in beats per minute (beats.min^−1^). The monitor was placed around the participant’s chest with an elastic strap and aligned with the sternum at the level of the inferior border of the pectoralis muscles. Functional strength (lower limb power) was measured pre- and post- training, using the countermovement jump. Players had to stand upright with their feet between 12.5 – 25.0 cm apart. On their dominant side, a white A1 sheet had been positioned on a wall next to them. While in this standing position, each participant was then asked to place black paint on their middle finger and stretching their arm up as high as they could, to touch the white sheet at their highest point - their standing reach height. They then had to move into a squat position and jump up as high as they could in one explosive movement. While airborne, they had to touch the white A1 sheet again at their highest point using their blackened middle finger. Countermovement jump measures were used to calculate muscle power based on a prediction formula (Equation 1) known as the Sayers equation:^[9]^


(Eq. 1) $$\begin{array}{l} \text{Peak power }(\text{watts})=60.7\times (\text{jump height }[\text{cm}])+45.3\times \\ (\text{body mass }[\text{kg}])-2055\\ Where\;jump\;height=height\;of\;jump-standing\;reach\;height \end{array}$$

#### Perceptual measures

Perceived exertion was assessed using a rating of perceived exertion scale developed by Borg.^[10]^ The scale ranges from a value of six, which represents perceptions of minimal exertion, to a value of 20, which represents maximal exertion. Players were required to focus on ‘Local’ rating of perceived exertion, as this provided an indication of perceived effort in the lower limb musculature, which included a single score specific to the quadriceps and hamstrings.

To assess perception of body discomfort, at the end of each over the Body Discomfort Scale and Map developed by Corlett and Bishop was utilised.^[11]^ Once the areas experiencing discomfort had been demarcated, a Likert scale on a scale from one to ten was used to rank the intensity of their discomfort. A value of one signified no discomfort and a value of ten represented extreme discomfort.

#### Performance measures

Ball release speed was measured using a Sports Radar Gun (SR 3600, Sports Radar Ltd, Florida, USA) placed directly behind the stumps at the non-strikers end. Accuracy was assessed using an accuracy board originally developed by Portus et al., which has three scoring zones (100, 50 and 25).^[12]^ The maximum scoring zone (100 points) rewarded balls that passed in line with the middle stump to approximately 25 cm outside off stump. The two other zones (50 and 25 points respectively) rewarded deliveries based on their impact point on the target, with 25 points being awarded to deliveries landing furthest away from the stumps within the target area. For deliveries that did not hit the target, a score of zero was awarded.

### Statistical analysis

Exploratory data analysis was conducted to examine data distributions, check for normality and identify outliers. Pre- and post-protocol data were compared using the non-parametric Wilcoxon signed-rank test. Changes in physiological, perceptual and performance measures over time were analysed using Kruskal Wallis tests, followed by post hoc Mann-Whitney U test with Bonferroni corrections. All data were analysed using Statistica™ version 7 software (Statsoft, South Africa, 2016). Significance was set at p < 0.05.

## Results

### Demographic, anthropometric and morphological parameters

A total of eight fast bowlers, with a mean age of 20 (± 2) years, height of 185.6 (± 7.7) cm, body mass of 78.6 (±13.9) kg, body mass index of 22.8 (±3.3) kg/m^2^ and a body fat percentage of 8.8 (±2.9) % participated in the study. Players participated in, on average, 3.6 days of moderate to high-intensity exercise per week.

### Physiological responses

**Fig. 1:**
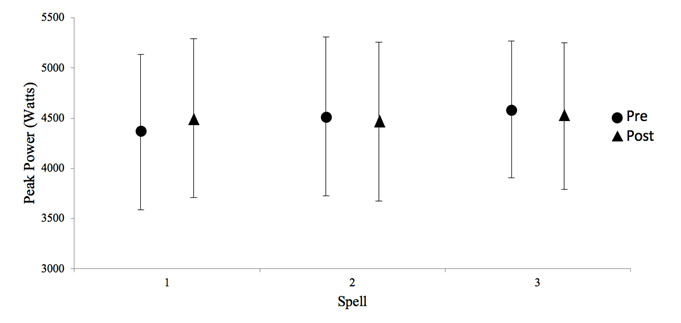
Mean (±SD) countermovement jump measures, pre- and post-spells.

Lower limb peak power was unchanged, pre- and post, in each bowling spell and there was no difference between spells (Fig. 1).

**Fig. 2:**
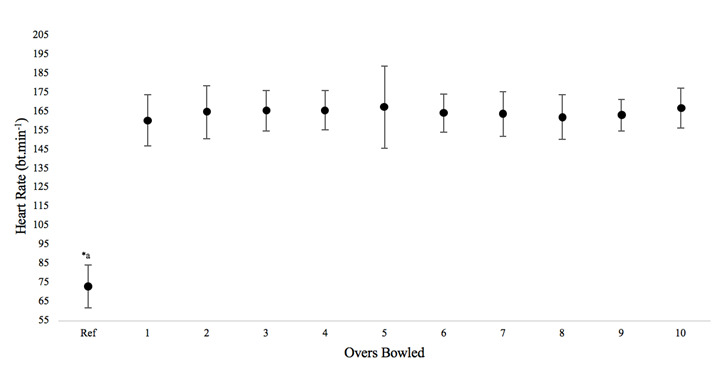
Mean (±SD) change in heart rate (beats.min^−1^) between each over bowled. a* indicates significant difference (p<0.05) between reference heart rate and all other heart rate measures.

Heart rate was significantly (p<0.05) elevated during all overs compared to the reference heart rate measured at the start of the protocol (Fig. 2). Overall, the heart rate remained constant throughout the bowling protocol. The lowest heart rate was observed following the first over (160 ± 14 beats.min^−1^). The highest was recorded following the fifth (167 ± 22 beats.min^−1^) and tenth (167 ± 11 beats.min^−1^) overs. A large inter-individual variability was evident following the fifth over (± 22 beats.min^−1^).

### Performance responses

**Fig. 3:**
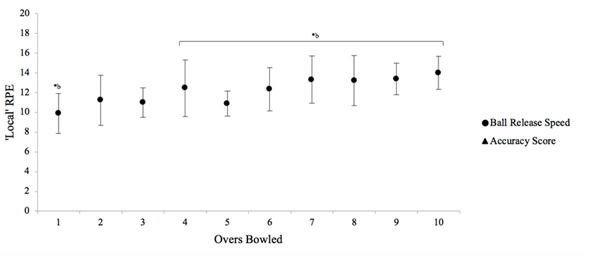
Mean (±SD) change in ball release speed (km.h-1) and accuracy score between each over bowled.

There was no change in either ball release speed or accuracy (Fig. 3). The lowest mean ball release speed was during the third over (90.81 ± 5.09 km.h^−1^) and the highest was during the tenth over (95.0 ± 7.6 km.h^−1^). The lowest mean accuracy score was in the third over (305.6 ± 80.8). The highest was in the ninth over (403.1 ± 94.0). A large inter-individual variability was apparent during overs six (± 124.7) and seven (± 177.7).

### Perceptual responses

**Fig. 4:**
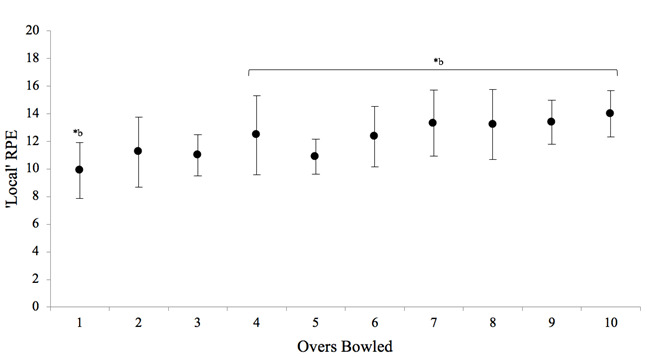
Mean (±SD) anterior and posterior perceptual ratings of body discomfort and frequency of rating for Spell 1, Spell 2 and Spell 3.

Body discomfort was most commonly experienced in the anterior and posterior shoulder areas of the bowling arm, the upper portion of the lower limb musculature, and the middle and lower back regions. Discomfort was also reported primarily on the posterior aspect of the body, particularly on the bowling arm side, lower back region and the lower limb musculature (Fig. 4). Frequency and relative intensity of discomfort in the shoulder regions remained largely unchanged throughout the three bowling spells. With regard to the back region, discomfort was observed in both the lower and middle back regions, with intensity increasing only marginally with each bowling spell. Contrastingly, a high number of players reported discomfort in their hamstrings, particularly of the non-leading leg. This discomfort increased in intensity throughout the three bowling spells. The frequency of reports for discomfort in the feet was greatest on the anterior side during the third spell, with moderate intensities being reported, particularly on the side of the leading leg.

**Fig. 5:**
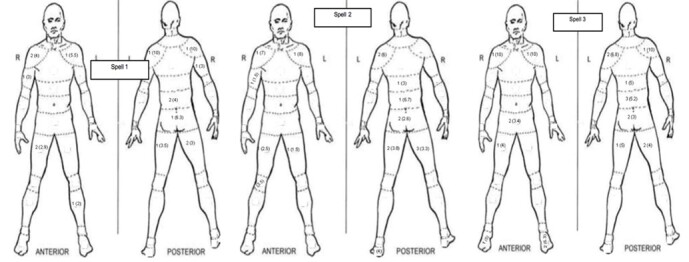
Mean (±SD) change in ‘local’ ratings of perceived exertion of the lower limb musculature (quadriceps and hamstrings) between each over bowled. *b indicates significant increases (p<0.05) in ‘local’ exertion ratings between overs one and four to ten.

Significant increases (p<0.05) in ‘local’ exertion ratings were evident between overs one and four to ten (Fig. 5). The lowest ‘local’ exertion rating was experienced at the end of the first over (10 ± 2) and the highest was at the end of the tenth over (14 ± 2).

## Discussion

The most important finding from this study was that performance was unchanged over the course of the bowling spell. This is the first study to show unchanged performance measures in an adolescent cohort. Studies done on non-adolescent bowlers have also shown unchanged performance with similar ball release speeds over time.^[3,12]^ This could be due to the fact that simulated protocols do not accurately reflect the demands experienced during real match situations.^[3]^ The only study which found a change in performance was a 12-over simulated protocol^[13]^ . However, this is extreme and not appropriate for adolescent players nor is it typical within a cricket game generally.^[13]^ While perceptions of effort and discomfort may pre-empt fatigue and injury risk, it is important to acknowledge the role of skill and mental aptitude, specifically the ability to concentrate on performance for a prolonged period of time.^[14]^ This can also impact performance measures as shown in previous studies.^[14]^ Although in other studies, accuracy was also unchanged, different accuracy measures have been used in different studies thus making comparisons difficult.^[3,12]^

Furthermore, lower limb power was unchanged which supports previous findings.^[3,13]^ Changes in strength are usually attributed to muscular fatigue which was not found in this study as a result of this protocol.^[2,5]^ By contrast, this study highlighted the local perception of strain experienced by bowlers, specifically in the hamstrings. This is an important finding as bowlers may perceive effort prior to changes in muscle power and/or strength which could possibly predispose them to injury even when other markers of fatigue remain intact (i.e. performance, lower limb power and heart rate). This finding needs to be investigated further as it could be a good indicator of the occurrence of potential injury. Heart rate reached steady state after a significant increase from the first over, indicating that exercise intensity remained similar for the remainder of the protocol. This is a finding which has been reported previously.^[3,13]^

## Conclusion

In conclusion, this study found that performance was consistent across a 10-over bowling spell and that lower limb muscle power remained unchanged. Heart rates reached steady state after the first over. By contrast, local ratings of perceived effort and body discomfort increased over time. This could mean that those unchanged measures do not accurately reflect fatigue or that perceptions are better indicators of imminent fatigue and potential injury risk. These authors had found previously that fast bowlers are the players most at risk of injury in cricket^[15]^.
